# Death of a Medical Man in a Snow Storm

**Published:** 1864

**Authors:** 


DEATH OF A MEDICAL MAN IN A SNOW STORM.—Dr. Gallice, practising in Langeac (Houte Loire), France, perished in the snow, on the 20th ult., whilst returning, on horseback, from his country rounds. He might have been saved but for the stupidity of some people, who feared to assist him without the aid of the rural police!

